# Mutant IDH1 Dysregulates the Differentiation of Mesenchymal Stem Cells in Association with Gene-Specific Histone Modifications to Cartilage- and Bone-Related Genes

**DOI:** 10.1371/journal.pone.0131998

**Published:** 2015-07-10

**Authors:** Yonghui Jin, Hassan Elalaf, Makoto Watanabe, Sakura Tamaki, Sho Hineno, Kazuhito Matsunaga, Knut Woltjen, Yukiko Kobayashi, Sanae Nagata, Makoto Ikeya, Tomohisa Kato, Takeshi Okamoto, Shuichi Matsuda, Junya Toguchida

**Affiliations:** 1 Department of Tissue Regeneration, Institute for Frontier Medical Sciences, Kyoto University, Kyoto, Japan; 2 Department of Cell Growth and Differentiation, Center for iPS Cell Research and Application, Kyoto University, Kyoto, Japan; 3 Department of Orthopaedic Surgery, Graduate School of Medicine, Kyoto University, Kyoto, Japan; 4 Life Science Research Center, Shimadzu Cooperation, Kyoto, Japan; 5 Department of Reprogramming Sciences, Center for iPS Cell Research and Application, Kyoto University, Kyoto, Japan; 6 Department of Gastroenterology and Hepatology, Graduate School of Medicine, Yamaguchi University, Ube, Japan; University of Wisconsin-Madison, UNITED STATES

## Abstract

Somatic mutations in the *isocitrate dehydrogenase* (*IDH)1/2* genes endow encoding proteins with neomorphic activity to produce the potential oncometabolite, 2-hydroxyglutarate (2-HG), which induces the hypermethylation of histones and DNA. The incidence of IDH1/2 mutations in cartilaginous tumors was previously shown to be the highest among various types of tumors, except for those in the central nervous system. Mutations have been detected in both benign (enchondromas) and malignant (chondrosarcomas) types of cartilaginous tumors, whereas they have rarely been found in other mesenchymal tumors such as osteosarcomas. To address this unique tumor specificity, we herein examined the effects of IDH1 R132C, which is the most prevalent mutant in cartilaginous tumors, on the differentiation properties of human mesenchymal stem cells (hMSCs). The induction of the *IDH1* R132C gene into MSCs markedly increased the amount of 2-HG and up-regulated global histone methylation. The induction of IDH1 R132C promoted the chondrogenic differentiation of hMSCs by enhancing the expression of *SOX9* and *COL2A1* genes in association with an increase in the active mark (H3K4me3), but disrupted cartilage matrix formation. On the other hand, IDH1 R132C inhibited expression of the *ALPL* gene in association with an increase in the repressive mark (H3K9me3), and subsequently inhibited the osteogenic properties of hMSCs and human osteosarcoma cells. Since osteogenic properties are an indispensable feature for the diagnosis of osteosarcoma, the inhibitory effects of IDH1 R132C on osteogenic properties may contribute to the lack of osteosarcomas with the *IDH1* R132C mutation. These results suggested that IDH1 R132C contributed to the formation of cartilaginous tumors by dysregulating the chondrogenic and osteogenic differentiation of hMSCs via gene-specific histone modulation.

## Introduction

Isocitrate dehydrogenases (IDH) are metabolic enzymes that catalyze the oxidative decarboxylation of isocitrate to α-ketoglutarate (α-KG), and consist of a gene family with three members: IDH1, IDH2 and IDH3, the first of which localizes in the cytoplasm while the latter two localize in mitochondria [1,2]. Somatic heterozygous *IDH1* or *IDH2* mutations have frequently been detected in glioma/glioblastomas by genome wide mutation searches [3,4]. Subsequent studies revealed that *IDH* mutations were extremely rare in primary (*de novo*) glioblastomas, but were common in recurrent glioblastomas developing secondary to low-grade tumors, which also frequently have IDH mutations [5]. These findings suggested that *IDH* mutations are an early event in gliomagenesis and persist during progression to recurrent glioblastomas.

*IDH1* mutations typically result in substitutions at codon R132, whereas *IDH2* mutations affect codon R172 or R140 [1,2]. Although a number of different mutants have been identified to date, the most common and important feature of mutant proteins is their neomorphic enzyme activity, which converts α-KG to 2-hydroxyglutarate (2-HG) [6]. Since 2-HG and α-KG are structurally identical, except that the C2 carbonyl group in α-KG is replaced by a hydroxyl group in 2-HG, 2-HG competes with α-KG and inhibits various α-KG-dependent enzymes including Jumonji-C domain-containing histone demethylase [7], the ten-eleven translocation (TET) family of 5-methylcytosine hydroxylases [8], and prolyl hydroxylase domain-containing proteins (PHD) [6]. These inhibitory effects induce aberrant DNA and histone methylation [7], and stabilize hypoxia inducible factor-1α, which then induces angiogenesis by up-regulating the VEGF gene [6]. Due to this pleiotropic function for dysregulating biological events, 2-HG is regarded as an oncometabolite that exerts tumor-inducing actions, and searches for *IDH* gene mutations have been performed in various types of malignancies [1,2]. *IDH1/2* mutations have consequently been detected in acute myeloid leukemia (AML) [9,10] and myelodysplastic disorders [11], but rarely in thyroid [12], prostate, B cell lymphoma, and colorectal carcinomas [13].

In 2011, cartilaginous tumors were added to the list of tumors with *IDH1* mutations [14,15] as tumors with the highest frequency *IDH* mutations, except for central nervous system tumors [16]. Cartilaginous tumors have been defined as tumors that produce cartilage-like tissues and consist of benign (such as enchondroma) and malignant (including conventional chondrosarcoma) tumors [17]. *IDH* mutations were found in both tumors with equal frequency [16], suggesting the role of *IDH* mutants in the initial step of transformation; however, the precise role of these mutants currently remains unknown. Most cartilaginous tumors, either benign or malignant, develop from the intramedullary region, and tumor cells have a chondrocyte-like morphology [17]. These clinical findings suggest that cells in bone marrow that have the ability to differentiate into chondrogenic cells are precursors of this type of tumor. Mesenchymal stem cells (MSCs) are defined as cells with differentiation properties for osteo-, adipo-, and chondrogenic lineages, and reside among bone marrow stromal cells [18], which are, therefore, reasonable candidates as the precursor cells of cartilaginous tumors.

We herein investigated the role of mutant IDH1 in the development of cartilaginous tumors using MSCs. We found that IDH1 mutants modified the differentiation properties of MSCs as well as the histone methylation of cartilage- and bone-related genes in a gene-specific manner. These results provide a novel insight into the role of IDH mutants in the development of cartilaginous tumors.

## Materials and Methods

### Ethics statement

The experimental protocols dealing human subjects were approved by the Ethics Committee of the Department of Medicine and Graduate School of Medicine, Kyoto University. Written informed consent was provided by each donor.

### Tissue specimens

Tumor tissue samples were obtained from patients with enchondromas (3 cases from Maffucci syndrome, 5 from Ollier’s disease, and 15 from solitary cases), chondrosarcomas (54), osteochondromas (7), and osteosarcomas (29) via either biopsy or resection in Kyoto University Hospital. Histological analyses were performed in all the samples, and showed that more than 90% of each tissue was composed of tumor cells.

### DNA extraction and direct DNA sequencing

High molecular weight genomic DNA was isolated from tumor tissues by standard phenol-chloroform extraction methods. DNA was amplified by PCR using two primer sets for exon 4 of the *IDH1* gene and exon 4 of the *IDH2* gene. The sequences of the primers used to detect *IDH* mutations have been reported previously [16]. PCR products were then sequenced on a 3500XL Genetic Analyzer (Applied Biosystems, Forester City, CA) with the Big Dye Terminator v3.1 Cycle Sequencing Kit (Applied Biosystems) according to standard procedures.

### Primary cultured cells

Bone marrow-derived MSCs from three donors (BM01, BM02, and BM03) were isolated with centrifugation and adhered to plastic dishes [19]. MSCs were cultured in α-minimal essential medium with GlutaMAX (Life Technologies, Carlsbad, CA) supplemented with 10% fetal bovine serum (HyClone., South Logan, UT, USA), 100 U/ml penicillin, and 100 mg/ml streptomycin. Expanded cells were characterized with cell surface markers and differentiation abilities as MSCs (Data not shown). Cells passaged less than five times were used in experiments.

### Establishment of the pLenti6-*IDH1* construct and lentiviral infection

The entire coding region of the human *IDH1* gene (NM_005896) with the N-terminal Flag and HA tag was cloned into the pDONR221 entry vector (Life Technologies). The *IDH1* R132C mutant was generated using site-directed mutagenesis. Therefore, primers were created and used on wild type *IDH1* cDNA in pDONR221. The wild type and R132C mutant of *IDH1* were transferred into the pLenti6/V5-DEST gateway vector (Life Technologies) via the LR-reaction, resulting in pLenti6-Flag-HA-*IDH1* (wt or R132C). The lentivirus was generated by transfecting 293FT cells with Lipofectamine 2000 (Life Technologies). hMSCs were infected with the lentivirus supernatant and selected with 5 μg/ml blasticidin (Invivogen, San Diego, CA).

### Intracellular metabolite extraction and quantification of 2-HG with GC-MS

Cultured cells were washed twice with ice-cold 0.9% NaCl aqueous solution. These cells were quenched with 1.5 ml of 80% methanol aqueous solution and chilled at -20°C for 5 min. They were then scraped off, resuspended in 80% methanol aq., and transferred to sample tubes. After centrifugation at 9,000×g for 5 min, the supernatant was collected, the cell pellet was resuspended in 0.5 ml of 80% methanol aq., and centrifuged at 9,000×g for 5 min. The supernatants were pooled and dried with SpeedVac. The pellets were dissolved with 80 μl of pyridine (Wako Pure Chemical Industries, Ltd., Osaka, Japan). After the addition of 40 μl of MSTFA (N-Methyl-N-TMS-Trifluoroacetamide) (GL Science Inc., Tokyo, Japan), the samples were incubated for 30 min at 30°C. The samples were centrifuged at 3,000×g for 5 min and the supernatants were subjected to a Gas chromatography-Mass spectrometry (GC-MS) analysis.

The GC-MS analysis was performed with GCMS QP2010 Ultra (Shimadzu Corporation, Kyoto, Japan). One microliter of the sample was injected with an auto injector AOC-20i (Shimadzu Corporation). After an initial 2 min at 80°C, the temperature was increased to 147°C at a rate of 15°C/min, followed by an additional constant temperature period at 330°C for 5 min. Gas chromatography was performed with a DB-5 column (30 m×0.25 mm I.D.) (Agilent Technologies, CA). Full scan mass spectra were acquired from m/z 85 to 460.

### Establishment of the osteosarcoma cell line with the doxycycline inducible *IDH1* gene by the PiggyBac transposon system

The entire coding region of the human *IDH1* gene was cloned into the pDONR221 entry vector. The *IDH1* R132C mutant was generated using site-directed mutagenesis. The wild type and R132C mutant of *IDH1* were transferred into KW111/GW, a derivative of PB-TET containing the rtTA transactivator [20], via the LR-reaction, resulting in KW111-*IDH1* (WT or R132C). One microgram of each KW111-*IDH1* and PBaseII plasmid DNA was co-transfected into human osteosarcoma cells (ANOS) [21] using the FuGENE HD transfection reagent (Promega, Tokyo, Japan). After being selected with G418 (500 μg/ml), drug-resistant cells were used as a bulk population, and the expression of each *IDH* gene was induced by doxycycline (0.2 μg/ml).

### Cell proliferation assay

A cell proliferation assay was performed using Cell Counting Kit-8 (Dojindo, Kumamoto, Japan). Cells were plated on 96-well plates at 1000 cells per well and cultured in growth medium. On days 1 and 8, 100 μl of growth medium with 10% CCK-8 solution was added to the wells, and the plate was incubated for a further 3 hours. Cell numbers in triplicate wells were then measured as the O.D. at 450 nm of reduced CCK-8.

### Western blotting

Western blotting was performed as described [22]. The primary antibodies used were as follows: #8137 for IDH1 (Cell Signaling Technology, Danvers, MA), #07–473 for H3K4me3, and #07–449 for H3K27me3, #07–690 for pan H3 (Millipore, Billerica, MA), and ab8898 for H3K9me3 (Abcam, Tokyo, Japan). Blots were probed with HRP-conjugated goat anti-mouse IgG or goat anti-rabbit IgG (Cell Signaling).

### Reverse transcription (RT) and quantified PCR (qPCR)

Total RNA was isolated from cells with the RNeasy Mini Kit (QIAGEN, Valencia, CA) and 1 μg of total RNA was used in the RT reaction with the SuperScript III first-strand synthesis system (Life Technologies) according to the manufacturer`s instructions. The primers used in qPCR were listed in S1 Table. qPCR was performed in triplicate using THUNDERBIRD SYBR qPCR Mix (QPS-201, TOYOBO, Japan).

### Induction of differentiation

Osteogenic differentiation was induced in growth medium supplemented with 0.1 μM dexamethasone, 50 μM ascorbic acid, and 10 mM β-glycerophosphate as previously described [22]. After a 14-day induction, calcium deposits were visualized by alizarin red staining, and the calcium content was quantified based on the OCPC method.

Chondrogenic differentiation was induced in the pellet culture (2.5× 10^5^ cells/pellet) with hMSC Chondrocyte Differentiation Medium (PT-3003, Lonza, Tokyo, Japan) supplemented with 10 ng/ml TGFβ3 for 21 days in a 15-ml centrifuge tube (#430791, Corning, NY, USA) or Lipidure-Coat 96-well plate (#81100525, Thermo Scientific, Yokohama, Japan), as previously described [22].

### Chromatin immunoprecipitation (ChIP) assay

A ChIP assay was performed as previously described [21]. Briefly, cross-linking was achieved by incubating cells in growth medium with formaldehyde at a final concentration of 1% for 10 min at room temperature. The protein-DNA complex was then extracted by lysis buffer (1% SDS; 10 mM EDTA; 50 mM Tris-HCl) and sonicated to shear DNA into 300–500 bp fragments. After centrifugation, the supernatants were incubated with antibodies at 4°C overnight. The next day, the complexes were precipitated by incubating with Protein G beads (Millipore) before centrifugation. After several washing steps, the chromatin-antibody complex was eluted with elution buffer (1% SDS, 0.1 M CH_3_CO_2_Na, 10 mM DTT), and DNA was separated from proteins with 200 mM NaCl at 65°C overnight, and subsequently with 50 μg/ml protein K at 45°C for 1 hr. DNA was purified with phenol/chloroform extraction and ethanol precipitation. The promoters or enhancer region were amplified by qPCR and mean enrichment relative to input was computed. The following primers were used to amply the *SOX9* promoter [23] (Accession No.: NG_012490): 5`-TTTCC ATTGA CTCCC TTTGC-3`and 5`-TGCCT GCAAA AGTGC TTAGA-3`, the *COL2A1* promoter [24] (Accession No.: NG_008072): 5`-CTGCT CCTTT CTACC GCTTT-3`and 5`-AGTTC TGCCG GAGTT GGAG-3`, the *COL2A1* enhancer [25] (Accession No.: NG_008072): 5`-GCTCC TCCGT CCACA CCT-3`and 5`-GGCTC GCTCA CAGAC ACC-3`, and the *ALPL* promoter [26] (Accession No.: NG_008940): 5`-GGTCC CCTTC TGCTT CTTCT-3`and 5`-CGTCT CTTTG TCTGC CTTCC-3`.

### Knock-down of *SOX9* with small interfering RNAs (siRNAs)

Two siRNAs against the *SOX9* gene, siSOX9#1 (s13307, CGCTCACAGT- ACGACTACAtt) and siSOX9#2 (s13308, AGCCCGATCTGAAGAAGGAtt), were obtained from Silencer Select Pre-designed siRNA (Life Technology) and transfected into IDH1 R132C hMSCs (5×10^5^ cells) using Lipofectamine RNAiMAX (Life Technologies). Silencer Select Negative Control siRNA #1 (#4390843) was used as a control.

### Statistical analysis

All results are the average of three independent experiments and are presented as the mean ± SE; results shown as the average of technical replications are presented as the mean ± SD. *P* values were calculated using the Student`s *t*-test or Dunnett multiple comparison test, and *p* values ≤0.05 were considered significant.

## Results

### Mutation spectrum of *IDH1/2* genes in bone tumors of Japanese patients

To obtain the *IDH1/2* mutation spectrum in our samples, 77 cartilaginous tumors, 7 osteochondromas, and 29 osteosarcomas were analyzed by direct sequencing. Three types of mutations caused three types of mutant *IDH1* (R132C, R132G, and R132H) genes while four types of mutations causing three types of mutant *IDH2* (R172S, R172T, and R172W) genes in cartilaginous tumors (S1 Fig); however, no mutations were detected in osteochondromas or osteosarcomas (Tables 1 and Table 2).

**Table 1:** Mutation spectrum of IDH1/2 genes in cartilaginous tumors.

Gene	Type of mutations	Base change	No. of tumors with mutations (%)
IDH1	R132C	CGT>TGT	16 (44.4)
IDH1	R132G	CGT>GGT	7 (19.4)
IDH1	R132F	CGT>CTT	2 (5.6)
IDH2	R172S	AGG>AGC	4 (11.1)
IDH2	R172S	AGG>AGT	4 (11.1)
IDH2	R172T	AGG>ACG	2 (5.6)
IDH2	R172W	AGG>TGG	1 (2.8)

**Table 2:** Mutation spectrum of IDH1/2 genes in each type of cartilaginous tumor.

Diagnosis		No. of tumors with mutation /No. of analyzed tumors (%)	Type of mutant (No. of tumor with each mutant)
Enchondroma	Maffucci syndrome	1/3(33.3)	R132C(1)
	Ollier’s disease	4/5(80.0)	R132C(3)
			R132G(1)
	Solitary	10/15(66.7)	R132C(6)
			R132G(5)
			R132H(2)
			R132S(2)
Chondrosarcoma		20/54(37.0)	R132C(9)
			R132G(2)
			R172S(6)
			R172T(2)
			R172W(1)

Of these mutations, 69% were detected in the *IDH1* gene and 31% in the *IDH2* genes. Furthermore, *IDH1* R132C was the most frequent mutation in this series. This mutation spectrum was consistent with previous findings [16].

### 2-HG produced by *IDH1* R132C induced histone methylation in hMSCs

**Fig 1:**
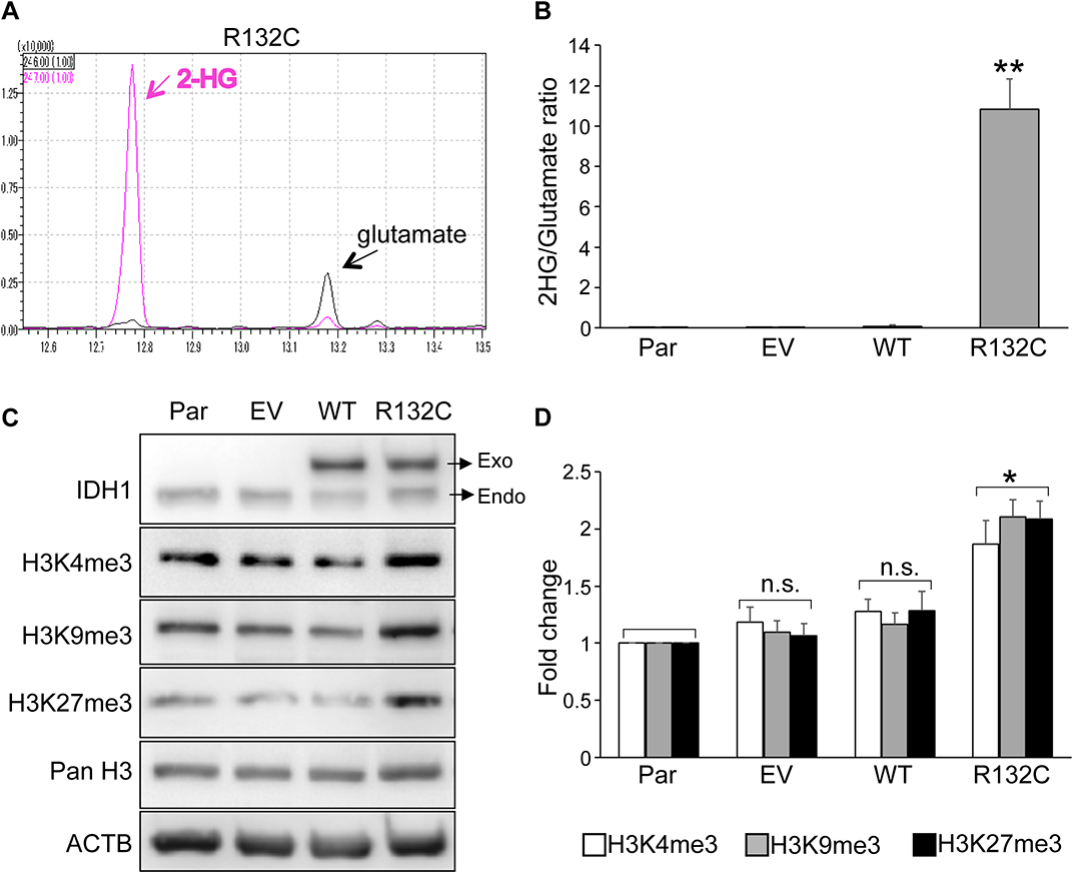
IDH1 R132C produced 2-HG and increased global histone methylation in hMSCs. **A.** Detection of 2-HG by GC-MS. Intracellular 2-HG was detected in the extracts of BM01 cells expressing the *IDH1* R132C gene. Peaks with *m/z* 246 and 247 were selected as quantification ions for glutamate and 2-HG, respectively. **B.** Relative amount of 2-HG in hMSCs. The amount 2-HG and glutamate was measured by GC-MS in cell extracts from each type of cell and the ratio was demonstrated. The mean ± SE from the results of three donors was shown. **C.** Expression of active (H3K4me3) and repressive (H3K9me3 and H3K27me3) histone marks. Protein lysates were prepared from each type of BM01 cell and used for western blotting to detect exogenous (Exo) and endogenous (Endo) IDH1 and indicated histone H3. **D.** Quantitative analyses of active and repressive histone marks. The expression level of each mark in infected hMSCs (EV, WT, or R132C) was demonstrated as a value relative to those in parental hMSCs (Par). The mean ± SE from the results of three donors was shown. Par, parental hMSC; EV, hMSCs infected with the empty vector; WT and R132C, hMSCs infected with the vector containing the wild-type *IDH1* or *IDH1* R132C gene, respectively. *, *p*<0.05 and **, *p*<0.01 by Dunnett`s multiple comparisons test compared to the parental cells.

Since the R132C mutant of *IDH1* was the most prevalent mutant in cartilaginous tumors, we decided to investigate its effects on the differentiation properties of hMSCs. Lentiviral expression vectors containing the wild type or *IDH1* R132C gene were introduced into each of three primary hMSCs. No significant difference was observed in the growth properties of transfected hMSC between wild-type IDH1 and IDH1 R132C, whereas both significantly affected the growth properties of hMSCs (S2 Fig). The GC-MS analysis confirmed the production of 2-HG in BM01 cells expressing *IDH1* R132C (Fig 1A). Similar results were obtained in the other two hMSCs (data not shown), and hMSCs expressing *IDH1* R132C produced markedly larger amounts of 2-HG than cells expressing the exogenous wild-type *IDH1* gene, in which the relative amount of 2-HG was equal to that in parental or cells infected with the empty vector (Fig 1B). To confirm the effects of 2-HG produced by *IDH1* R132C, global histone methylation was analyzed by western blotting using antibodies against each type of methylated histone (Fig 1C). The amounts of active (H3K4me3) and repressive marks (H3K9me3 and H3K27me3) were increased in cells expressing *IDH1* R132C, whereas no significant differences were observed in cells expressing wild-type exogenous *IDH1* (Fig 1D). These results indicated that infected *IDH1* R132C exerted its function to produce 2-HG and subsequently induced histone methylation.

### *IDH1* R132C enhanced the expression of chondrocyte-related genes, but disturbed pellet formation after chondrogenic differentiation

**Fig 2:**
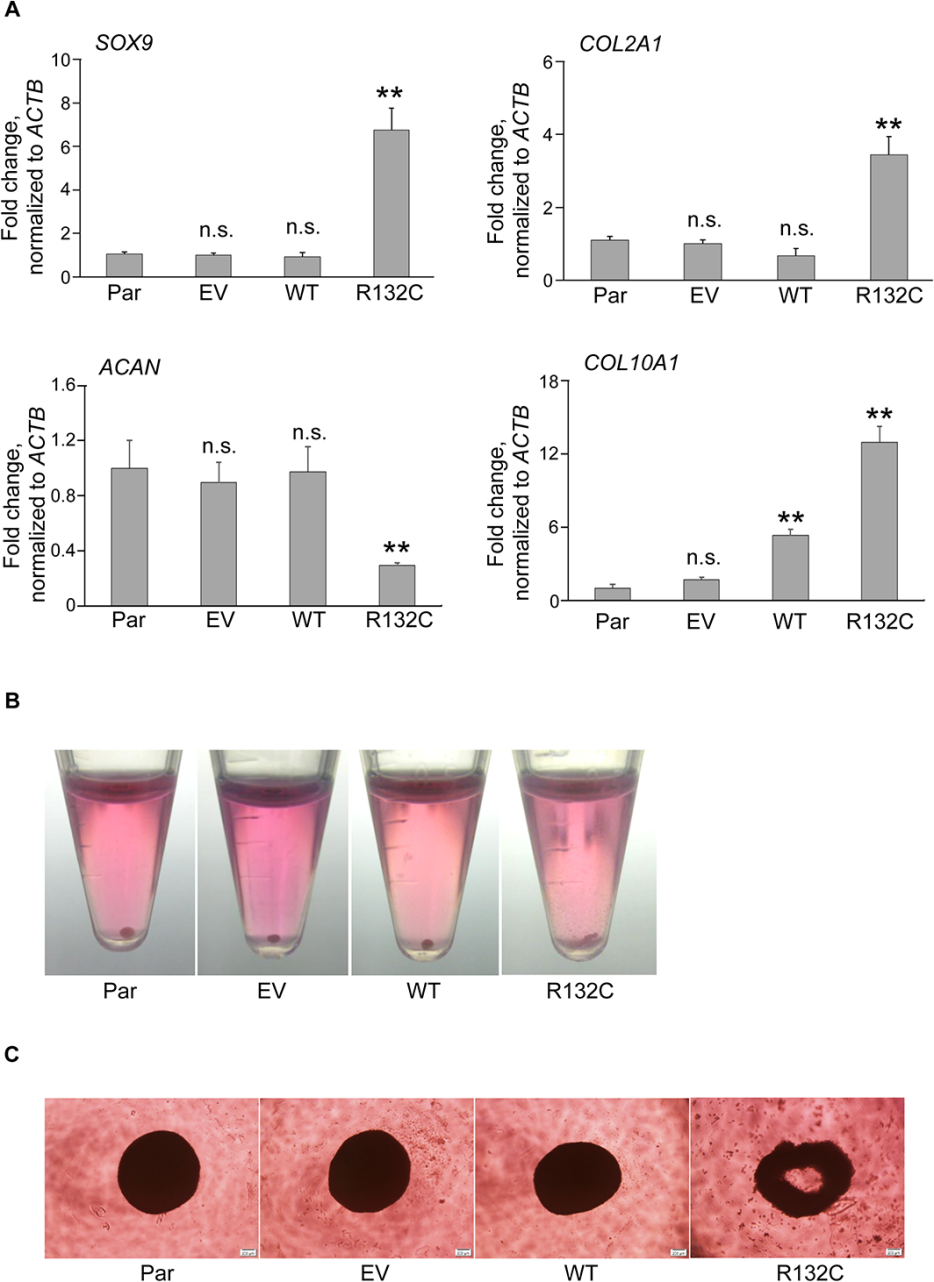
The effect of IDH1 R132C on chondrogenic properties of hMSCs. **A.** The mRNA expression of *SOX9*, *COL2A1*, *ACAN*, and *COL10A1* genes in hMSCs. RNAs were extracted from each hMSC and analyzed by qRT-PCR. Data were expressed as the mean value relative to that of parental cells after normalizing to expression of the *ACTB* gene. Results are the mean ± SE from the results of three different donors Representative photos of pellet formation after chondrogenic induction in 15 ml centrifuge tube **(B)** and in ultralow adhesion 96-well plates (**C**). **, *p*<0.01 by Dunnett`s multiple comparisons test compared to the parental cells. Par, EV, MT, and R132C were as described in the legend for Fig 1.

Since *IDH* gene mutations were preferentially observed in cartilaginous tumors, we investigated the effects of *IDH1* R132C on the expression of cartilage-related genes in hMSCs (Fig 2). Surprisingly, without the induction of chondrogenic differentiation, the expression levels of the *SOX9* gene, a master transcription factor for chondrogenesis, was up-regulated in hMSCs expressing *IDH1* R132C (Fig 2A). The expression of *COL2A1* and *COL10A1* genes, which encode the major extracellular matrix (ECM) protein in cartilage, was also up-regulated (Fig 2A). As for the genes encoding other ECM proteins, the expression of the *ACAN* gene was down-regulated (Fig 2A), whereas that of the *COMP* gene remained unchanged (data not shown).

To evaluate the differentiation property of these hMSCs, 3-dimensional chondrogenic differentiation assays were performed, which showed hMSCs expressing *IDH1* R132C failed to form tight cartilaginous pellets. All 10 samples prepared from parental, empty vector-, and wild type *IDH1*-transduced BM01 cells successfully created tight pellets, whereas none of the 10 samples derived from BM01 cells expressing *IDH1* R132C showed pellets (Fig 2B). Similar results were obtained using ultralow adhesion plates, which showed the failure of *IDH1* R132C cells to make a spheroidal structure (Fig 2C). Identical results were obtained in the other two hMSCs (data not shown), suggesting that *IDH1* R132C disturbed the ability of cells to make cartilaginous matrix.

### *IDH1* R132C inhibited the expression of *ALPL* gene and disturbed mineralization after osteogenic induction

**Fig 3:**
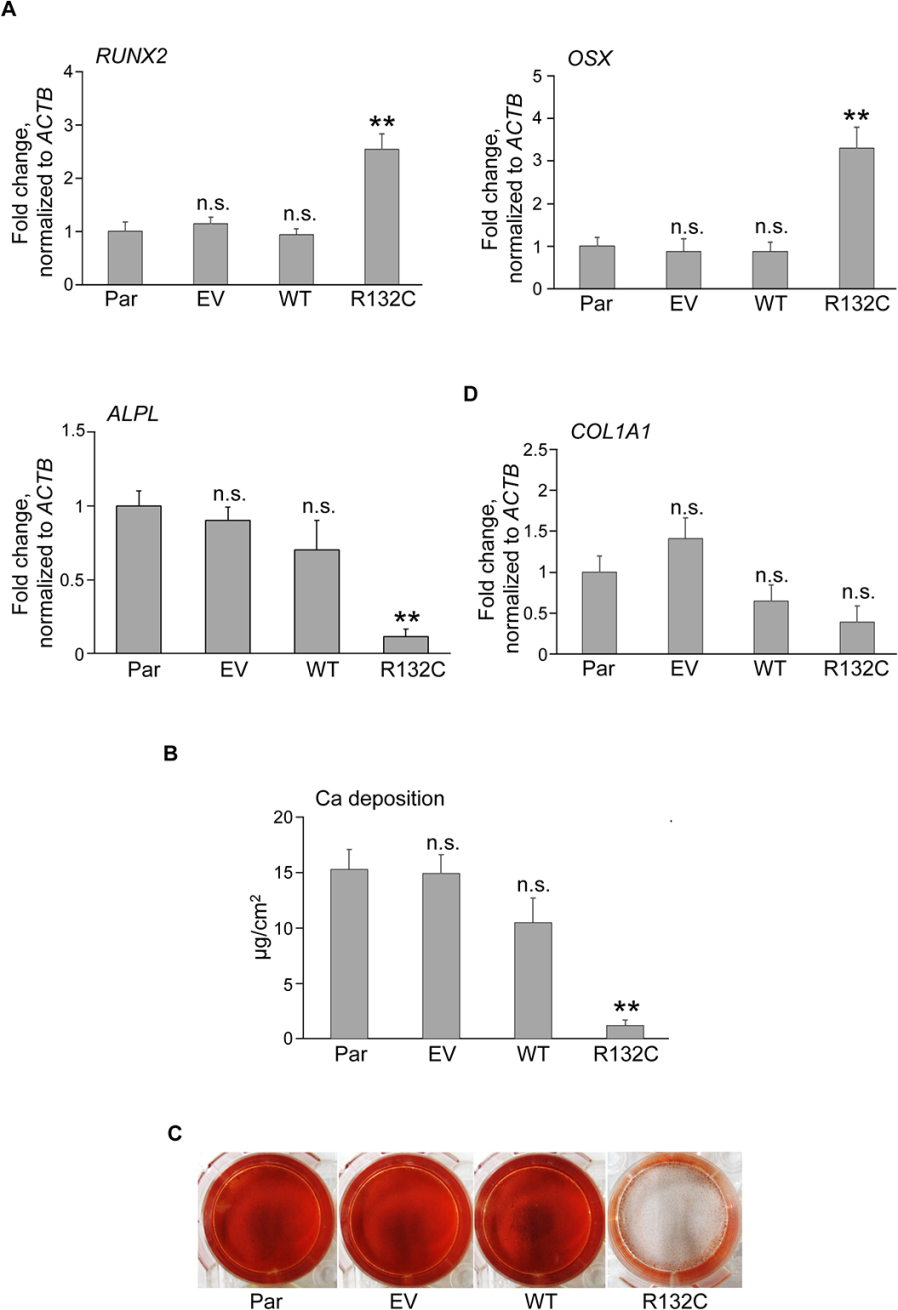
The effect of IDH1 R132C on osteogenic properties of hMSCs. **A.** The mRNA expression of *RUNX2*, *OSX*, *ALPL* and *COL1A1* genes in hMSCs. RNAs were extracted from each hMSC and analyzed by qRT-PCR. Data are shown as a value relative to that of parental cells after normalizing to expression of the *ACTB* gene. **B.** Calcium (Ca) deposition after osteogenic induction in each hMSC. Data were normalized to the culture area. Results (**A** and **B**) are the mean ± SE from the results of three different donors. **C.** Calcified nodule formation after osteogenic induction. Each type of BM01 cell was cultured under osteogenic induction conditions and then stained with Alizarin Red S. **, *p*<0.01 by Dunnett`s multiple comparisons test compared to the parental cells. Par, EV, MT, and R132C were as described in the legend for Fig 1.

Then we examined the effects of *IDH1* R132C on the osteogenic differentiation in hMSCs. The expression of *RUNX2* and *Osterix* genes was up-regulated in hMSCs by the introduction of *IDH1* R132C (Fig 3A). However, the expression of *alkaline phosphatase*, *liver/bone/kidney (ALPL)* gene was markedly down-regulated in hMSC expressing *IDH1* R132C (Fig 3A). ALPL is a key enzyme in bone formation because it plays a critical role in the mineralization of the extracellular matrix [27].

Consistent with this reduction in the expression of the *ALPL* gene, hMSCs expressing the *IDH1* R132C gene failed to form calcified nodules stained with Alizarin red (Fig 3B) and deposited markedly lower amounts of Ca (Fig 3C) after the osteogenic induction, suggesting that *IDH1* R132C inhibited the osteogenic properties of hMSCs.

**Fig 4:**
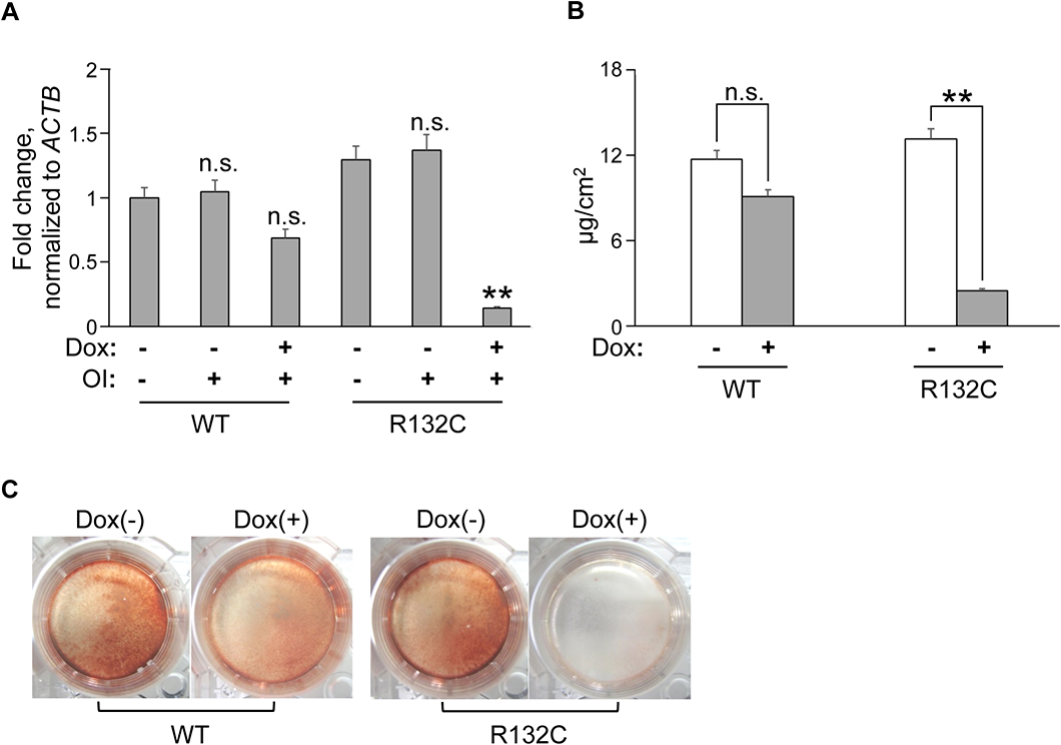
IDH1 R132C inhibited the osteogenic differentiation of human osteosarcoma cells. **A.** mRNA expression of the *ALPL* gene. RNAs were extracted from ANOS cells transduced with doxycycline (Dox) inducible expression vectors containing the wild-type *IDH1* (WT) or *IDH1* R132C (R132) gene before and after osteogenic induction (OI) and analyzed by qRT-PCR. Data are shown as a value relative to that of WT-ANOS cells before OI after normalizing to expression of the *ACTB* gene expression. **, *p*<0.01 by Dunnett`s multiple comparisons test compared to the WT cells or R132C cells without Dox treatment. **B.** Calcium (Ca) deposition after osteogenic induction in WT- and R132C-ANOS cells. Data were normalized to the culture area. **, *p*<0.01 by the Student`s *t*-test. **C.** Calcified nodule formation after osteogenic induction. WT- or R132C-ANOS cells were cultured under osteogenic induction conditions with or without doxycycline and then stained with Alizarin Red S. Error bars indicate the average ± SD from three biological replicates.

This inhibitory effect was analyzed further in an osteosarcoma cell line with the inducible *IDH1* gene. The induction of *IDH1* R132C, but not wild-type *IDH1*, down-regulated the expression of the *ALPL* gene (Fig 4A), and osteogenic differentiation properties were inhibited, as determined by the lower amount Ca deposited (Fig 4B) and smaller number of Alizarin-red-positive calcified nodules (Fig 4C), which was consistent with the results obtained in hMSCs.

### *IDH1* R132C differently regulated the expression of chondrocyte- and osteocyte-related genes by diverse histone modification

**Fig 5:**
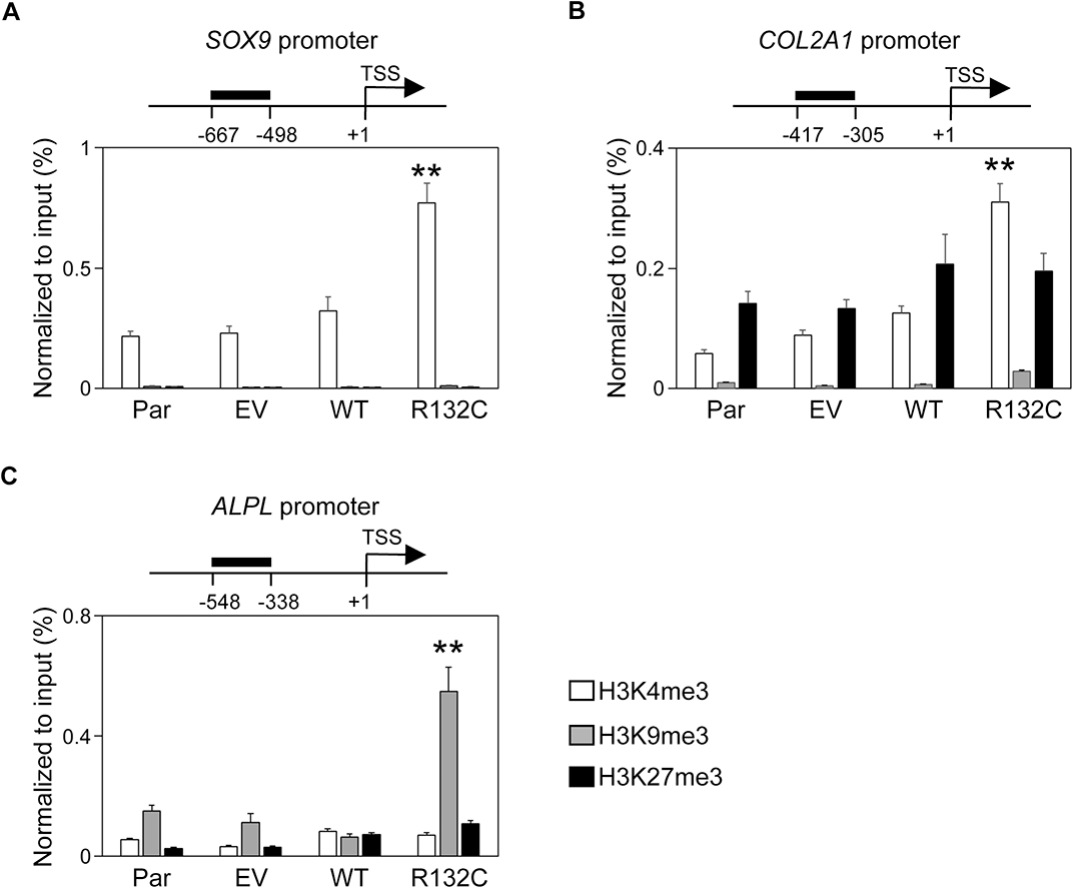
IDH1 R132C differentially regulated the expression of chondrocyte- and osteocyte-related genes by gene-specific epigenetic modulation. Active and repressive histone marks associated with the promoter regions of the *SOX9* (**A**), *COL2A1* (**B**), and *ALPL* (**C**) genes. The status of histone modification in each hMSC (Par, EV, MT, or R132C, as described in the legend for Fig 1) was analyzed by chromatin immunoprecipitation (ChIP) using antibodies against H3K4me3, H3K9me3, and H3K27me3. The target region of each locus was shown in the scheme at the top of each graph. Data were presented by qPCR, and the values were indicated relative to the input. Error bars reflect SD in 3 experiments. TSS, transcription start site. **, *p*<0.01 by Dunnett`s multiple comparisons test compared to the parental cells.

To investigate whether these effects of *IDH1* R132C on differentiation were related to its effects on histone methylation, histone modifications in the promoter regions of *SOX9*, *COL2A1*, and *ALPL* genes were investigated. Histones associated with the promoter region of the *SOX9* gene in parental hMSCs were mainly modified with the active mark (H3K4me3) and less with the repressive marks (H3K9me3 and H3K27me3). The induction of *IDH1* R132C markedly increased the amount of H3K4me3; however, the effects on H3K9me3 or H3K27me3 were hardly detected (Fig 5A). Similar results were observed for the promoter region of the *COL2A1* gene (Fig 5B), in which both active and repressive (H3K27me3) marks was observed in parental cells. Although the induction of *IDH1* R132C increased the amount of both marks, a significant difference was only observed for the active mark. The results for the enhancer region of the *COL2A1* gene also showed the preferential up-regulation of the active mark (S3 Fig). The up-regulation of the *COL2A1* gene remained unchanged even when the expression of the *SOX9* gene, its major regulator, was inhibited by siRNA (S4 Fig), suggesting that epigenetic modifications contributed significantly to its expression. In contrast, the effects of *IDH1* R132C on the *ALPL* gene were completely different. Although both active and repressive marks were detected in the promoter region of the *ALPL* gene in parental cells, only the repressed mark (H3K9me3) was increased by the expression of *IDH1* R132C (Fig 5C). These effects of *IDH1* R132C on histone modification in each gene correlated well with the effects on the expression level of each gene, suggesting that the effects of *IDH1* R132C on the expression of three genes and subsequently on differentiation properties were via epigenetic modifications.

## Discussion

Certain metabolites have critical roles as regulators and co-factors of important enzymes in various biological pathways, and recent cancer research disclosed that metabolic alterations contributed to the initiation and development of malignant cells. 2-HG produced by mutant IDH proteins is a prototype of these oncometabolites, and a number of studies have disclosed the role of 2-HG in malignant transformation [1,2].

One interesting and still unanswered question regarding *IDH* mutations in cancer is their tumor-type-specific prevalence. Mutations in *IDH* genes are the most frequent genetic abnormality in central nervous system (CNS) tumors, but are extremely rare in other types of cancers such as prostate and colorectal cancers [1,2]. Cartilaginous tumors frequently share mutations in *IDH* genes with these in CNS tumors [16], even though these two tumor types are histologically and developmentally different. This incidence may be related to many factors, one of which may be their effects on differentiation through epigenetic modifications. Impaired differentiation by mutant *IDH* has been reported in CNS, hematopoietic, and hepatocholangial differentiation processes [28–30]. Therefore, we herein examined the effects of *IDH1* R132C on the differentiation properties of hMSCs, which presumably are precursors of cartilaginous tumors. hMSCs with *IDH1* R132C began to express the early (*SOX9*) and late (*COL2A1* and *COL10A1*) chondrogenic markers (Fig 2). After chondrogenic induction, however, cells expressing *IDH1* R132C failed to make cartilage-like pellets (Fig 2). The hydroxylation of proline residues is an important step for collagen maturation, which reinforces its helical structure and promotes its thermal stability [31]. This reaction was catalyzed by prolyl-4-hydroxylases, which are regulated by α-KG and, therefore, inhibited by 2-HG [1,2]. A previous study demonstrated that the maturation of type IV collagen was inhibited in IDH1 R132H knock-in mice, resulting in fragile basement membranes [32], which may be relevant to our results on the cartilage matrix. Mutations in the *COL2A1* gene itself have been identified in approximately one third of chondrosarcomas [33], which may also impair the maturation of type II collagen. The down-regulated expression of the *ACAN* gene, another important ECM protein, by *IDH1* R132C may also have contributed to the fragile structure of cell pellets (Fig 2). Therefore, although cartilaginous tumors are defined as tumors that produce cartilage-like tissues, the matrix produced may be immature, which may help tumor cells to proliferate and invade surrounding tissues.

The introduction of an *IDH2* R172K mutation, the main *IDH2* mutation in gliomas, into C3H10T1/2 was previously shown to reduce the expression of the *COL2A1* gene and gave rise to poorly differentiated sarcomas in xenograft models [34]. We currently cannot explain the reason for this discrepancy. In order to determine whether this was caused by differences in donor cells, we introduced *IDH1* R132C into C3HT101/2 cells, and found that *IDH1* R132C up-regulated the expression of the Col2a1 gene in mouse cells (data not shown). Therefore, this difference may be related to the type of mutation (*IDH1* R132C versus *IDH2* R172K), which may affect enzymatic activity, as reported previously [35].

The specific occurrence of *IDH* mutations in cartilaginous tumors among mesenchymal tumors is another issue that needs to be clarified [16]. Although osteosarcomas are the most frequent primary malignant bone tumors, no patients with *IDH1* mutations have been described in previous and current studies on osteosarcomas. Osteosarcomas and chondrosarcomas share some clinical phenotypes: both develop from bone marrow in most cases and are diagnosed by the presence of a specific extracellular matrix. They also share some genetic abnormalities such as chromosomal aneuploidy and alteration in the Rb and p53 pathways [33,36]. However, the incidence of *IDH* mutations clearly differs.

We demonstrated that *IDH1* R132C induced the expression of early markers, the *RUNX2* and *OSX* genes, whereas that of the *ALPL* gene was markedly inhibited, and is the key enzyme initiating ECM mineralization. Osteogenic properties are one of most important diagnostic features of osteosarcomas [17], and, therefore, the inhibitory effect of *IDH1* R132C on osteogenic differentiation may interfere tumor cells to be diagnosed as osteosarcoma.

The gene-specific effects of *IDH1* R132C was an interesting finding. *IDH1* R132C increased histone methylation in both cartilage- and bone-related genes as well as global histone methylation. However, target modifications, either active or repressive, markedly differed in a gene-specific manner, and ultimately had different effects on the expression of each gene. We currently have no data to explain the mechanism underlying this gene-specific regulation, but consider this an important issue for understanding the effects of *IDH* mutations on the differentiation of various types of cancers.

## Supporting Information

S1 Fig *IDH1/2* mutations in cartilaginous tumors. Three types of *IDH1* mutations (R132C, R132G, R132H) and three types of *IDH2* mutations (R172S, R172T, R172W) were detected in 77 cartilaginous tumors.
(PDF)

S2 Fig The proliferation of hMSCs expressing *IDH1* WT or R132C. Each type of hMSC was plated in 96-well plates at 1000 cells per well. On day 1 and 8, the cell numbers in triplicate wells were measured as the absorbance at 450 nm of reduced CCK-8. *, *p*<0.05 by Dunnett`s multiple comparisons test compared to the parental cells. Par, EV, WT, and R132C were as described in legend for Fig 1.
(PDF)

S3 Fig Epigenetic modifications to histones associated with the enhancer region of the *COL2A1* gene. Active and repressive histone marks associated with the enhancer region of the *COL2A1* gene in each hMSC were analyzed by ChIP using the antibodies against H3K4me3, H3K9me3, and H3K27me3. The target region was shown in the scheme at the top of each graph. Data were presented by qPCR, and the values were indicated relative to the input. Error bars reflect SD in 3 experiments. TSS, transcription start site. **, *p*<0.01 by Dunnett`s multiple comparisons test compared to the parental cells. Par, EV, WT, or R132C were as described in the legend for Fig 1.
(PDF)

S4 Fig Knock-down of the *SOX9* gene in *IDH1* R132C hMSCs. hMSCs expressing *IDH1* R132C were treated with siRNA targeting *SOX9* for 48 hours, and the expression of the *SOX9* gene as well as the *COL2A1* gene was analyzed by qPCR. Two different siRNAs targeting the *SOX9* gene were used. **, *p*<0.01 by Dunnett`s multiple comparisons test compared to the n.c (negative control) cells.
(PDF)

S1 Table Primer sequences used in qRT-PCR. (PDF)
