# Supplementary material for: Mutant IDH1 Dysregulates the Differentiation of Mesenchymal Stem Cells in Association with Gene-Specific Histone Modifications to Cartilage- and Bone-Related Genes
Source: PLoS One. 2015 Jul 10;10(7):e0131998. doi: 10.1371/journal.pone.0131998 (PMC4498635; doi:10.1371/journal.pone.0131998)
Supplement: S1 Fig — Three types of IDH1 mutations (R132C, R132G, R132H) and three types of IDH2 mutations (R172S, R172T, R172W) were detected in 77 cartilaginous tumors. (PDF) [file pone.0131998.s001.pdf]

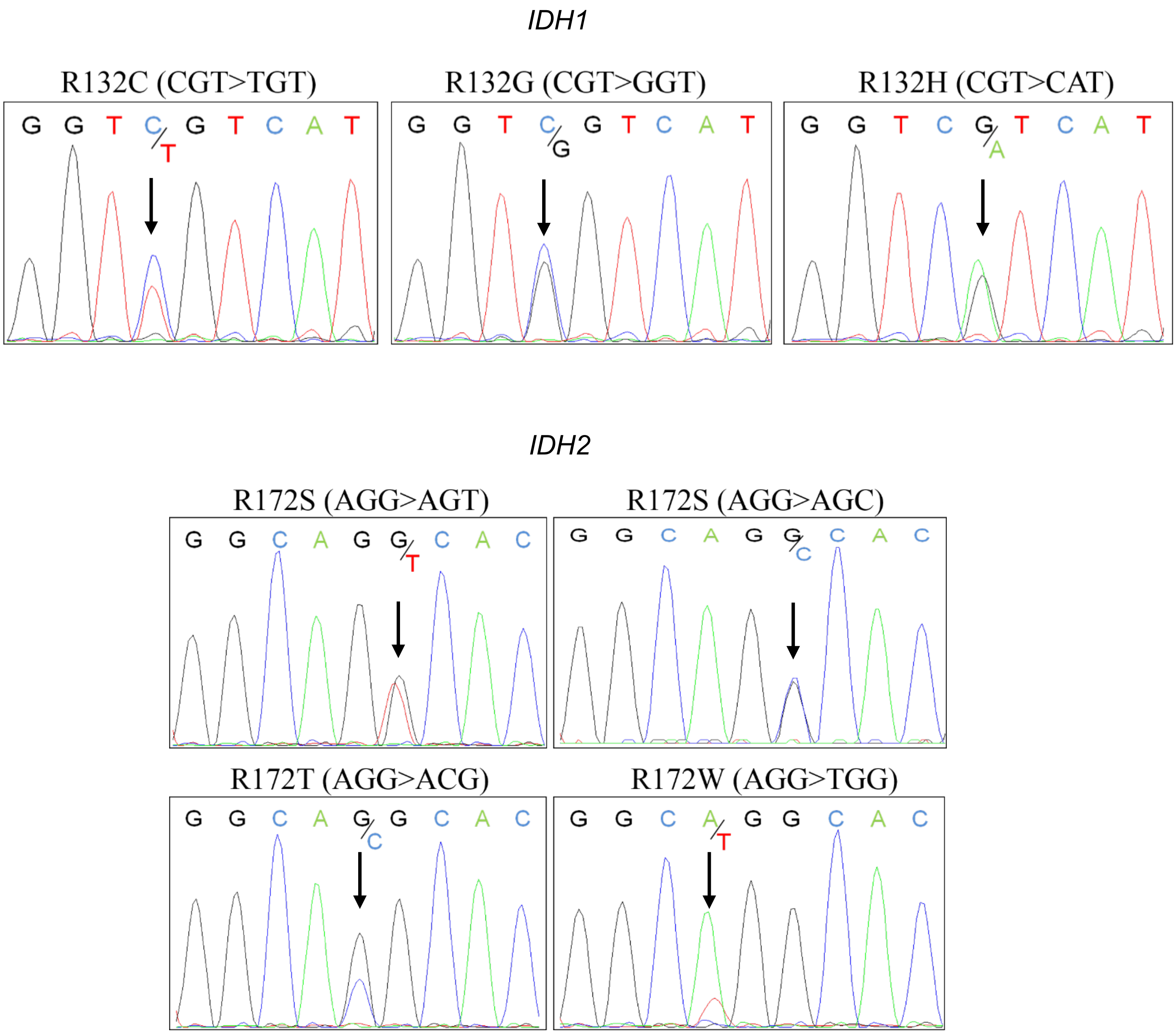


**Figure S1. *IDH1/2* mutations in cartilaginous tumors.**
Three types of *IDH1* mutations (R132C, R132G, R132H) and three types of *IDH2* mutations (R172S, R172T, R172W) were detected in 77 cartilaginous tumors.
