# Supplementary material for: Mutant IDH1 Dysregulates the Differentiation of Mesenchymal Stem Cells in Association with Gene-Specific Histone Modifications to Cartilage- and Bone-Related Genes
Source: PLoS One. 2015 Jul 10;10(7):e0131998. doi: 10.1371/journal.pone.0131998 (PMC4498635; doi:10.1371/journal.pone.0131998)
Supplement: S2 Fig — Each type of hMSC was plated in 96-well plates at 1000 cells per well. On day 1 and 8, the cell numbers in triplicate wells were measured as the absorbance at 450 nm of reduced CCK-8. *, p<0.05 by Dunnett`s multiple comparisons test compared to the parental cells. Par, EV, WT, and R132C were as described in legend for Fig 1. (PDF) [file pone.0131998.s002.pdf]

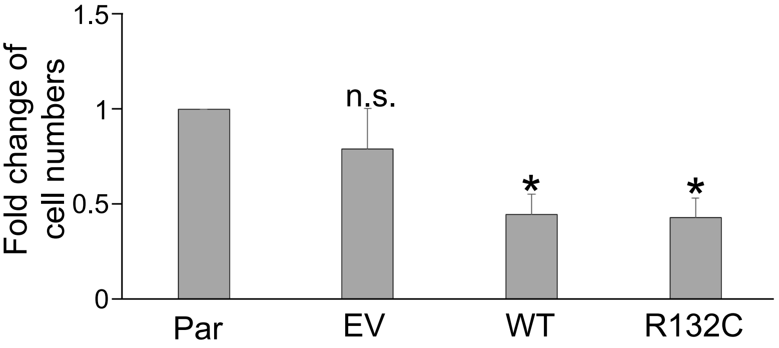

Figure S2. The proliferation of hMSCs expressing *IDH1* WT or R132C. Each hMSC (Par, EV, WT, or R132C, as described in legend for Figure 1) were plated in 96-well plates at 1000 cells per well. At 1 day and 8 days after plating, the cell numbers in triplicate wells were measured as the absorbance at 450 nm of reduced CCK-8. The fold change of cell numbers was calculated by fold change of the absorbance in 7 days. Data are shown as a value related to that of parental cells. *, $p<0.05$, by Dunnett`s multiple comparisons test compared to the parental cells.
