# Supplementary material for: Mutant IDH1 Dysregulates the Differentiation of Mesenchymal Stem Cells in Association with Gene-Specific Histone Modifications to Cartilage- and Bone-Related Genes
Source: PLoS One. 2015 Jul 10;10(7):e0131998. doi: 10.1371/journal.pone.0131998 (PMC4498635; doi:10.1371/journal.pone.0131998)
Supplement: S3 Fig — Active and repressive histone marks associated with the enhancer region of the COL2A1 gene in each hMSC were analyzed by ChIP using the antibodies against H3K4me3, H3K9me3, and H3K27me3. The target region was shown in the scheme at the top of each graph. Data were presented by qPCR, and the values were indicated relative to the input. Error bars reflect SD in 3 experiments. TSS, transcription start site. **, p<0.01 by Dunnett`s multiple comparisons test compared to the parental cells. Par, EV, WT, or R132C were as described in the legend for Fig 1. (PDF) [file pone.0131998.s003.pdf]

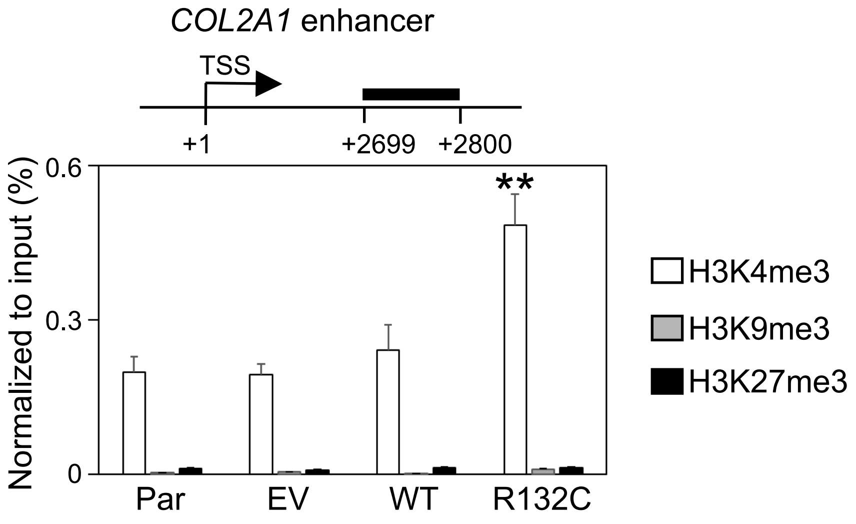

Figure S4. Epigenetic modifications to histones associated with the enhancer region of the *COL2A1* gene.
Active and repressive histone marks associated with the enhancer region of the *COL2A1* gene in each hMSC (Par, EV, WT, or R132C, as described in the legend for Figure 1) were analyzed by chromatin immunoprecipitation (ChIP) using the antibodies against H3K4me3, H3K9me3, and H3K27me3. The target region was shown in the scheme at the top of each graph. Data were presented by qPCR, and the values were indicated relative to the input. Error bars reflect SD in 3 experiments. TSS, transcription start site. **$P<0.01$, by Dunnett`s multiple comparisons test compared to the parental cells.
