# Supplementary material for: Mutant IDH1 Dysregulates the Differentiation of Mesenchymal Stem Cells in Association with Gene-Specific Histone Modifications to Cartilage- and Bone-Related Genes
Source: PLoS One. 2015 Jul 10;10(7):e0131998. doi: 10.1371/journal.pone.0131998 (PMC4498635; doi:10.1371/journal.pone.0131998)
Supplement: S4 Fig — hMSCs expressing IDH1 R132C were treated with siRNA targeting SOX9 for 48 hours, and the expression of the SOX9 gene as well as the COL2A1 gene was analyzed by qPCR. Two different siRNAs targeting the SOX9 gene were used. **, p<0.01 by Dunnett`s multiple comparisons test compared to the n.c (negative control) cells. (PDF) [file pone.0131998.s004.pdf]

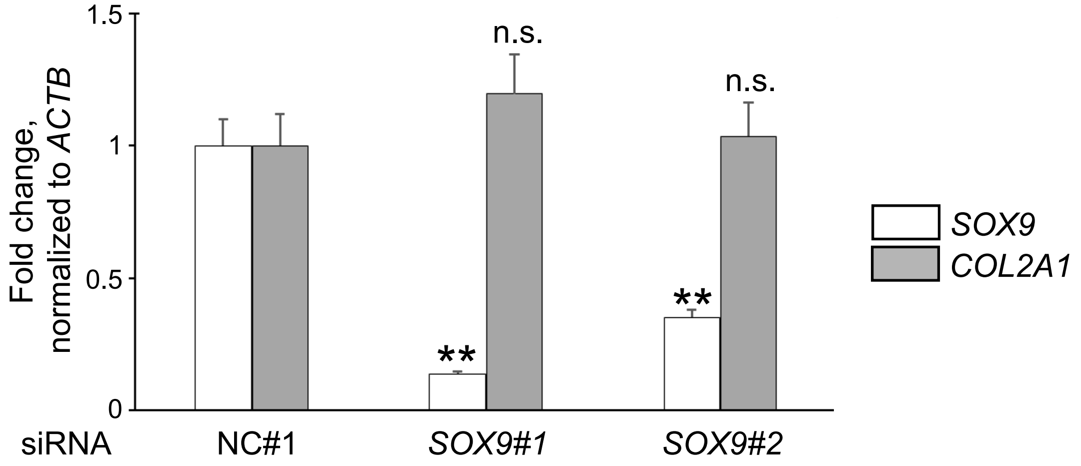

S4. The knock-down of *SOX9* gene in *IDH1* R132C hMSCs.
hMSCs expressing *IDH1* R132C were treated with siRNA targeting the *SOX9 gene* for 48 hours, and the expression of the *SOX9* gene as well as the expression of *COL2A1* gene was analyzed by qPCR. Two different siRNA targeting the *SOX9* gene were used. ＊＊, p<0.01 by Dunnett's multiple comaprisons test compared to cells treated with control siRNA (NC#1)
