# Supplementary material for: Mutant IDH1 Dysregulates the Differentiation of Mesenchymal Stem Cells in Association with Gene-Specific Histone Modifications to Cartilage- and Bone-Related Genes
Source: PLoS One. 2015 Jul 10;10(7):e0131998. doi: 10.1371/journal.pone.0131998 (PMC4498635; doi:10.1371/journal.pone.0131998)
Supplement: S1 Table — (PDF) [file pone.0131998.s005.pdf]

Table S1. Primer sequences used in qRT-PCR

Target gene	Accession No.	Primer Sequences		product size
		Sense	Antisense	
SOX9	NM_000346	AGCAAAGGAGATGAAATCTGTTCTG	AGGTTAACTGCTGGTGTTCTGAGA	151bp
COL2A1	NM_001844	GGCAATAGCAGGTTCACGTACA	CGATAACAGTCTTGCCCCACTT	79bp
ALPL	NM_000478	CCTCCTCGGAAGACACTCTG	GCAGTGAAGGGCTTCTTGTC	139bp
COMP	NM_000095	AGGGAGATCGTGCAGACAA	AGCTGGAGCTGTCCTGGTAG	154bp
ACAN	NM_013227	TGAGTCCTCAAGCCTCCTGT	TGGTCTGCAGCAGTTGATTC	129bp
COL10A1	NM_000493	AATGCCCACAGGCATAAAAG	AGGACTTCCGTAGCCTGGTT	187bp
RUNX2	NM_001024630	TTACTTACACCCCGCCAGTC	TATGGAGTGCTGCTGGTCTG	139bp
OSX	NM_001173467	GCCAGAAGCTGTGAAACCTC	GCTGCAAGCTCTCCATAACC	161bp
COL1A1	NM_000088	GTGCTAAAGGTGCCAATGGT	ACCAGGTTCACCGCTGTTAC	128bp
